# The National Neurology Forum 2023: Patients, Doctors, Authorities, Industry - Together for the Future of Neurology

**DOI:** 10.25122/jml-2023-1020

**Published:** 2023-05

**Authors:** Stefana-Andrada Dobran, Alexandra Gherman, Dafin Muresanu

**Affiliations:** 1. RoNeuro Institute for Neurological Research and Diagnostic, Cluj-Napoca, Romania; 2. Sociology Department, Babes-Bolyai University, Cluj-Napoca, Romania; 3. Department of Neuroscience, Iuliu Hatieganu University of Medicine and Pharmacy, Cluj-Napoca, Romania

**Figure 1:**
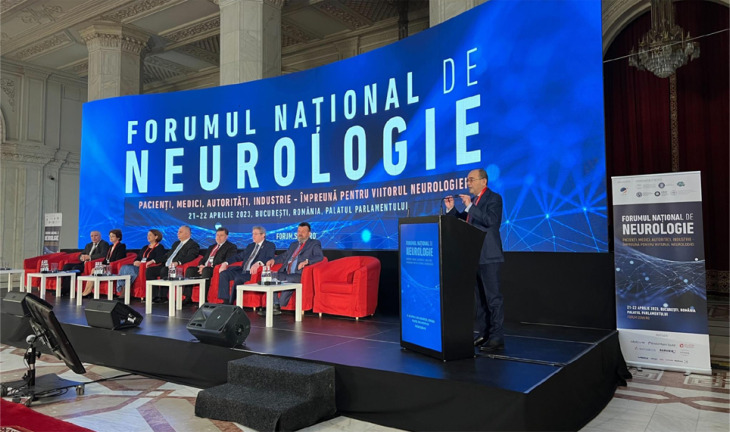
The National Neurology Forum, 21-23 April 2023

The National Neurology Forum (NNF), held on April 21-22, 2023, at the Palace of Parliament in Romania, served as a platform for exchanging ideas and presenting advancements in the field of neurology (Figure 1). This editorial provides a comprehensive overview of the event, highlighting key discussions, outcomes, and potential implications for the future of neurology at the national level and beyond. The event was marked by the presence of esteemed professionals and thought leaders who shared their insights and experiences, contributing to the rich tapestry of discussions and debates. This year's NNF was a true milestone for the Romanian neurological community, marking the convergence of minds and ideas aimed at advancing the understanding and treatment of neurological disorders, serving as a testament to the commitment and dedication of the Romanian Society of Neurology to improving patient care and outcomes.

The event highlighted the National Strategy for Combating Cardiovascular and Cerebrovascular Diseases (SNBCC) as its central focus. Announced by Health Minister Prof. Dr. Alexandru Rafila in the autumn of 2022 and developed under the aegis of the Romanian authorities, the SNBCC is an initiative aligned with the 2023-2030 National Health Strategy and European level initiatives such as the Stroke Action Plan for Europe (SAP-E). Cardiovascular and cerebrovascular diseases are the leading cause of mortality and morbidity in the Romanian population, having a significant economic burden on the healthcare system. The SNBCC aims to implement a systematic reform plan for the management of healthcare practices at the national level to ultimately improve population health. The strategy will help policymakers to improve care for the Romanian population by implementing a comprehensive reform plan in the field of cardiovascular and cerebrovascular diseases, based on a detailed assessment of their impact on the Romanian population, which will inform initiatives focusing on prevention, investments in medical staff training, infrastructure, increasing access to neurorehabilitation, and consolidating the national network for acute stroke care.

The forum was chaired by Prof. Dr. Dafin Muresanu (EFNR President), Prof. Dr. Cristina Tiu (President of the Romanian Neurology Society), and Prof. Dr. Bogdan O. Popescu (Vice Rector at the University of Medicine and Pharmacy Carol Davila Bucharest). Supported by the Foundation of the Society for the Study of Neuroprotection and Neuroplasticity (SSNN), the Ministry of Health, the Romanian Society of Neurology (SNR), Carol Davila University of Medicine and Pharmacy, Iuliu Hatieganu University of Medicine and Pharmacy, and George Emil Palade University of Medicine, Pharmacy, Science and Technology, the event featured an array of topics, such as neurology, neurosurgery, cardiology, diabetes and nutritional diseases, and physical and rehabilitation medicine. Officials from public authorities, universities of medicine and pharmacy, and national and international professional societies were present at the scientific proceedings.

Panel discussions covered a range of topics, including the implications of SNBCC for the Romanian healthcare system, the importance of prevention in cardiovascular and cerebrovascular diseases, Parkinson's disease, epilepsy, cognitive impairment and dementia, multiple sclerosis, peripheral neuropathies, neuro-oncology, headache, and migraine. Each panel provided comprehensive approaches to understanding and addressing the various aspects of neurological conditions and healthcare. Discussions also emphasized the need for enhanced collaboration among the private and public sectors and authorities, converging towards the common goal of improved education and communication of public health knowledge.

A multidisciplinary panel on prevention underscored the critical role of tackling risk factors early when managing cardiovascular and cerebrovascular diseases. The discussions revolved around the need for proactive measures for prevention and improvement of patient outcomes. The session featured presentations on the latest research in prevention strategies, followed by a panel discussion on the practical aspects of implementing these strategies in clinical practice and identifying solutions for enhanced collaboration among the private and public sectors, converging towards the common goal of improved education and communication of public health knowledge.

The Parkinson's Disease (PD) panel showcased perspectives on at-home care, the personal, clinical, and economic impact of the disease, and the objective evaluation regarding the burden of advanced PD. The session also highlighted patient and caregiver perspectives on the neurological degenerative condition and encompassed different approaches to the early identification of at-risk patients, improved access to care, and expanded education and medical and psychosocial care. Neurorehabilitation, as an integral part of PD patient care, was also discussed in terms of adequate neurological rehabilitation with multidisciplinary teams, the current state of medical infrastructure, and solutions to expand patient access to high-quality care.

The epilepsy panel approached the importance of prioritizing this disease within national action plans, highlighting the relevance of awareness campaigns and specialized services and emphasizing the scientific significance of recent progress in epilepsy research and innovation. Additionally, the panel provided perspectives on developing a national registry for epilepsy and pediatric neurological diseases and improving medical access in Romania's rural communities. The session aimed to promote the objectives of the Intersectoral Global Action Plan (IGAP) on epilepsy and other neurological disorders and the Brain Health Strategy (EAN) to reduce the burden of neurological diseases and improve the quality of life for patients worldwide.

The next panel centered around cognitive impairment and dementia, showcasing the importance of early identification and the emotional and social impact on patients and their families. Participants discussed emerging trends and progress in diagnosing and managing cognitive impairment, including innovative therapies and non-pharmacological interventions. The session highlighted the importance and benefits of multidisciplinary collaboration and partnerships among healthcare professionals, patients, and authorities to develop personalized strategies to improve patients' quality of life. Overall, the unique challenges of cognitive impairment and the need for innovative care strategies represented the central theme.

The multiple sclerosis panel discussed the burden of the disease and its impact on the patient's quality of life, emphasizing the role of integrative approaches in managing the symptoms and the impact of early identification, monitoring, and access to personalized therapies. The debates tackled the perspective of healthcare actors and stakeholders and were directed toward identifying the main challenges and solutions for raising patient care standards through collaborative efforts among patients, professionals, and healthcare authorities.

Focused discussions on peripheral neurological diseases followed, centered around the interdisciplinary approaches in the diagnosis and management of several disorders including Spinal Muscular Atrophy (SMA), Amyotrophic lateral sclerosis (ALS), progressive muscular dystrophy, Pompe disease, or amyloid neuropathy.

The experts discussed the importance of early diagnosis and treatment in neuro-oncological affections, pinpointing the need for comprehensive multidisciplinary approaches from the perspective of oncology, radiotherapy, neurosurgery, psycho-oncology, and palliative care. The use of minimally invasive and modern neurosurgery techniques was debated, as well as the importance of comprehensive evaluation in cerebral metastasis, immunohistochemistry, and pathologic diagnosis. Lastly, the session addressed the determination of tumoral mutations and the use of NGS technology, as well as off-label therapies, clinical studies, and genetic counseling.

The headache and migraine panel brought forward recent innovations in diagnosis and treatment and explored the international classification of headaches and migraines and their impact on patients’ lives. The session aimed to identify the most efficient solutions for patients’ treatment by offering a comprehensive approach to all the perspectives outlined.

The Romanian healthcare system stands to benefit from integrative approaches, better access to medical care, and increased awareness and education regarding cardiovascular and cerebrovascular diseases. The future of neurology and general healthcare should be built upon the collaborative action of all actors involved and center around improving resource allocation, patient care, and medical personnel training. Considering the current disparities in access to care for the population, broadly accessible approaches are direly needed.

**Figure 2:**
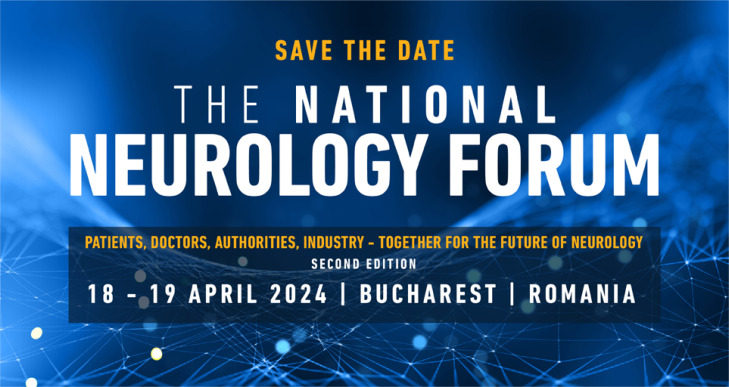
The National Neurology Forum 2024, second edition

To support and encourage reformative actions of the Romanian Healthcare System, the second edition of the National Neurology Forum will be organized next year, on 18-19 April 2024 (Figure 2). The following Forum aims to further understanding and multidisciplinary collaboration in managing neurological affections through fresh perspectives and interactive approaches and find development opportunities for the Romanian Healthcare System. We look forward to meeting you at the second edition of the National Neurology Forum to enhance collaborations on improving scientific knowledge and access to care, thus ensuring better health and care for the Romanian population!

